# Occupational Exposures, Chronic Obstructive Pulmonary Disease and Tomographic Findings in the Spanish Population

**DOI:** 10.3390/toxics12100689

**Published:** 2024-09-24

**Authors:** Eduardo Loeb, Jan-Paul Zock, Marc Miravitlles, Esther Rodríguez, Hans Kromhout, Roel Vermeulen, Juan José Soler-Cataluña, Joan B. Soriano, Francisco García-Río, Pilar de Lucas, Inmaculada Alfageme, Ciro Casanova, José Rodríguez González-Moro, Julio Ancochea, Borja G. Cosío, Jaume Ferrer Sancho

**Affiliations:** 1 Departamento de Medicina, Universitat Autònoma de Barcelona (UAB), 08193 Barcelona, Spain; eloebm@gmail.com (E.L.); esther.rodriguez@vallhebron.cat (E.R.); 2 Servicio de Neumología, Centro Médico Teknon, Grupo Quironsalud, 08022 Barcelona, Spain; 3 National Institute for Public Health and the Environment (RIVM), P.O. Box 1, 3720 BA Bilthoven, The Netherlands; jan-paul.zock@rivm.nl; 4 Servicio de Neumología, Hospital Universitari Vall d’Hebron, 08035 Barcelona, Spain; marcm@separ.es; 5 Centro de Investigación Biomédica en Red de Enfermedades Respiratorias (CIBERES), Instituto de Salud Carlos III (ISCIII), 28029 Madrid, Spain; jjsoler@telefonica.net (J.J.S.-C.); jbsoriano2@gmail.com (J.B.S.); fgr01m@gmail.com (F.G.-R.); juli119@separ.es (J.A.); borja.cosio@ssib.es (B.G.C.); 6 Vall d’Hebron Institut de Recerca (VHIR), Vall d’Hebron Barcelona Hospital Campus, 08035 Barcelona, Spain; 7 Institute for Risk Assessment Sciences, Utrecht University, 3584 CM Utrecht, The Netherlands; h.kromhout@uu.nl (H.K.); r.c.h.vermeulen@uu.nl (R.V.); 8 Julius Center for Health Sciences and Primary Care, University Medical Center Utrecht, P.O. Box 85500, 3508 GA Utrecht, The Netherlands; 9 Departamento de Medicina, Servicio de Neumología, Hospital Arnau de Vilanova-Lliria, Universitat de València, 46015 Valencia, Spain; 10 Departamento de Medicina, Servicio de Neumología, Hospital Universitario La Princesa, Universidad Autónoma de Madrid, 28006 Madrid, Spain; 11 Departamento de Medicina, Servicio de Neumología, Hospital Universitario La Paz-IdiPAZ, Universidad Autónoma de Madrid, 28029 Madrid, Spain; 12 Servicio de Neumología, Hospital General Gregorio Marañon, 28007 Madrid, Spain; pilar.delucas@gmail.com; 13 Departamento de Medicina, Unidad de Gestión Clínica de Neumología, Hospital Universitario Virgen de Valme, Universidad de Sevilla, 41014 Sevilla, Spain; inma.alfageme.michavila@gmail.com; 14 Departamento de Medicina, Servicio de Neumología-Unidad de Investigación Hospital Universitario Nuestra Señora de Candelaria, CIBERES, ISCIII, Universidad de La Laguna, 38010 Santa Cruz de Tenerife, Spain; casanovaciro@gmail.com; 15 Departamento de Medicina, Servicio de Neumología, Hospital Universitario Vithas Madrid Arturo Soria, Universidad Europea (UE), 28043 Madrid, Spain; respirama@yahoo.es; 16 Departamento de Medicina, Servicio de Neumología, Hospital Universitario Son Espases-IdISBa, Universidad de las Islas Baleares, 07120 Palma de Mallorca, Spain

**Keywords:** chronic obstructive pulmonary disease, COPD, occupational exposures, job exposure matrix, JEM, computed tomography, emphysema

## Abstract

Self-reported occupational exposure was previously associated with COPD in the Spanish population. This study aimed to analyse the relationship between occupational exposure to various chemical and biological agents, COPD, emphysema, and the bronchial wall area, which was determined by lung computed tomography (CT) in 226 individuals with COPD and 300 individuals without COPD. Lifetime occupational exposures were assessed using the ALOHA(+) job exposure matrix, and CT and spirometry were also performed. COPD was associated with high exposure to vapours, gases, dust and fumes (VGDF) (OR 2.25 95% CI 1.19–4.22), biological dust (OR 3.01 95% CI 1.22–7.45), gases/fumes (OR 2.49 95% CI 1.20–5.17) and with exposure to various types of solvents. High exposure to gases/fumes, chlorinated solvents and metals (coefficient 8.65 95% CI 1.21–16.09, 11.91 95%CI 0.46- 23.36, 14.45 95% CI 4.42–24.49, respectively) and low exposure to aromatic solvents (coefficient 8.43 95% CI 1.16–15.70) were associated with a low 15th percentile of lung density indicating emphysema. We conclude that occupational exposure to several specific agents is associated with COPD and emphysema in the Spanish population.

## 1. Introduction

Genetic and environmental factors are involved in the pathogenesis of chronic obstructive pulmonary disease (COPD), and among these, smoking is known to be the major risk factor. However, according to the definition of this disease, other inhaled agents may play a causal role [1,2]. In this regard, 20–45% of COPD patients are non-smokers [3]. Occupational exposure, environmental pollution, and exposure to biomass fumes have also been shown to be risk factors that should be taken into account [4,5].

The relationship between exposure to substances in the workplace and COPD has been described in different world populations [6,7]. In Spain, a recent study including more than 7500 subjects from the general population over 40 years of age showed that self-reported exposure to vapours, gases, dusts and fumes (VGDF) was associated with COPD [8] (odds ratio (OR) 1.22 95% confidence interval (CI) 1.03–1.44) with a population attributable fraction of 8.2%. Based on these results, several aspects should be considered to better understand the influence of occupational exposure on the development of COPD.

In studies based on self-reported exposure, the information depends on the response to a generic question asking about the existence or not of occupational exposure. However, detailed information on the different inhaled agents to which workers have been exposed is required to better understand the risk of developing COPD posed by the workplace. The best method to obtain this information is to make a comprehensive list of occupational activities, code the jobs according to a validated coding classification and apply a general population job exposure matrix (JEM) that allows the semi-quantitative estimation of worker’s exposure to different agents. Regarding study design, studies focused on the general population are useful for analysing the relationship between occupational exposure and COPD in order to minimise common biases in cohorts of workers, such as the survivor and healthy worker effect [9]. In this regard, different populations have been studied with inconsistent results. Thus, an association between occupational exposure and COPD has been detected in some studies [10,11,12,13] but not in others [14,15]. In a review of general population studies conducted using the ALOHA (+) JEM, only low exposure to mineral dust and exposure to gases/fumes were associated with COPD [16], whereas, in a later study, the association occurred for biological dusts, fumes/gases and pesticides [17]. This disparity of results suggests the need for further studies, especially in understudied populations, such as those in Southern Europe.

The relationship between occupational exposure and alterations of the lung parenchyma and bronchial airway is less well known. Chronic airflow obstruction, the hallmark of COPD, is caused both by emphysema, with decreased elastic retraction pressure of the lung, and small airway remodelling and obstruction [18]. These alterations are detectable by high-resolution computed tomography (CT) [19]. Indeed, the quantification of lung density, which is indicative of emphysema, has been applied in multiple studies [20], and the dimensions of the bronchi have also been determined [21,22].

In an initial study on smoking subjects, Marchetti et al. [23] detected that the percentage of emphysema and air trapping (small airway involvement) in CT was higher in subjects exposed to dust and fumes, while greater bronchial wall thickness was only detected in occupationally exposed men. 

In a second study, also on smokers, longer occupational exposure to VGDF was associated with increased emphysema and bronchiolar alterations [24]. In both studies, the exposure was self-reported and grouped under the term “VGDF”, precluding analysis by specific occupational agents.

The present study had two objectives: first, to analyse the relationship between occupational exposure defined by the ALOHA(+) JEM with COPD defined by spirometry and respiratory symptoms in the Spanish population, and second, we analysed the relationship between occupational exposure and lung morphology measured by thoracic CT.

## 2. Materials and Methods

### 2.1. Design

This study is included within the national, epidemiological, multicentre cross-sectional Spanish EPISCAN II study, the protocol, fieldwork and methods of which have been described previously [25,26]. Briefly, this study was conducted in 20 university hospitals across Spain from April 2017 to February 2019.

This study was approved by the Ethics Committees of each of the participating centres, and all participants gave informed consent to participate. The EPISCAN II protocol is registered at https://clinicaltrials.gov (NCT03028207) and at www.gsk-clinicalstudyregister.com/estudio/205932.

### 2.2. Study Subjects

The EPISCAN II study recruited a randomly selected sample, targeting subjects from the general population over 40 years of age from all the autonomous communities of Spain, stratified by zip codes, with quotas defined by sex and age groups. For the purposes of the present study, a subgroup of subjects for whom a thoracic CT scan was available was analysed. This test was performed on the first 35 subjects with COPD and the first 35 subjects without COPD in 12 of the participating centres, with the aim of recruiting approximately 400 individuals for each group. COPD was defined by a postbronchodilator FEV1/FVC less than 0.7. Each participant completed a structured questionnaire on work history, a questionnaire on respiratory symptoms, and forced spirometry.

### 2.3. Procedures and Measurements

#### 2.3.1. Study Variables

The demographic data of the subjects included were obtained, as well as information about smoking and educational level.

Pre-bronchodilator and post-bronchodilator spirometry were performed using a pneumotachograph (Vyntus Spiro, Carefusion, Germany), according to the recommendations of the Spanish Respiratory and Thoracic Surgery Society (SEPAR) [27], and using the Global Lung Initiative equations [28] as reference values.

The European Coal and Steel Community (ECCS) questionnaire on respiratory symptoms was administered to all subjects [29]. Biomass exposure was defined as an affirmative answer to the following question: have you had or have regular contact with smoke from wood or logs (e.g., fireplaces/wood stoves, work activity related to wood burning, etc.)?

Each subject was administered an occupational questionnaire adapted from the Spanish version of the European Community Respiratory Health Survey [14]; this included full occupational history of all jobs held during their working life. For each of these jobs, their occupation and industry were recorded as free text and subsequently coded according to the International Standard Classification of Occupations 1988 (ISCO-88) by one experienced coder [30]. Subsequently, occupational exposures were assessed by linking ISCO-88 occupational codes to the semiquantitative ALOHA(+) JEM [13,15]. For each occupational code, this JEM assigned three degrees of exposure intensity (none, low, and high) to 10 categories of agents: biological dust, mineral dust, gases/fumes, herbicides, insecticides, fungicides, aromatic solvents, chlorinated solvents, other solvents, and metals. Three composite exposure types from the above were also assigned (any pesticides; any solvents; and vapours, gases, dusts and/or fumes (VGDF)).

CT images were acquired during maximal inspiration, without contrast administration and with low radiation doses. The images obtained were subjected to semi-automated processing to determine the percentage of emphysema, areas of low attenuation, and bronchial tract wall thickness, as previously described [26]. Volumetric CT scans were taken at full inspiration using a first-generation dual system. Whole-lung images were extracted automatically, and the attenuation coefficient of each pixel was calculated. To assess parenchymal remodelling and quantitatively express morphological features, the following variables were chosen: (a) % emphysema volume defined by the percentage of a lung low attenuation area < −950 Hounsfield units (HUs) at full inspiration, and (b) 15th percentile of lung density, which is the HU threshold corresponding to the lowest 15% of lung attenuation (attenuation distribution percentile). To assess airway dimensions, we used two measurements: the lumen diameter and bronchial wall thickness of both primary and secondary bronchus, measured near the origin of two segmented bronchi. Therefore, the total bronchial area and lumen area could be estimated. The wall area (WA) was calculated as the total bronchial area minus the lumen area; the percentage of the wall area (%WA) resulted from the quotient between the WA/total bronchial area × 100 [31].

#### 2.3.2. Statistical Analysis

Categorical variables were presented as numbers with percentages, and continuous variables as the mean with standard deviation (SD).

Differences between groups were analysed using Chi-square and Student’s *t*-tests. Unconditional logistic regression models were used to evaluate the relationship between COPD and occupational exposures. Age, sex, educational level, smoking, cumulative exposure in pack-years and exposure to biomass were introduced as adjustment variables in each model. The associations detected were expressed as OR with their 95% CIs.

Analysis of variance and the Kruskal–Wallis test were used to evaluate the relationship between the CT characteristics, COPD, smoking status, and the matrix variables. In addition, multivariate linear regression analyses were performed to analyse the relationship between CT characteristics and matrix variables. These models were adjusted for age, sex, education, smoking, cumulative pack-year exposure, biomass exposure and the presence of COPD.

The SAS Enterprise Guide version 7.15 statistical package (SAS Institute, Cary, NC, USA) was used for data analysis. A *p*-value less than 0.05 was considered statistically significant in all analyses.

## 3. Results

### 3.1. Population

A total of 526 participants, with a mean age of 63 (SD: 11) years, were recruited, with 290 (55.1%) being females. Most clinical characteristics were significantly different in participants with and without COPD. In the COPD group, the mean age, proportion of males and tobacco use were significantly higher. Both the forced expiratory volume in one second (FEV_1_) and the FEV_1_/forced vital capacity (FVC) ratio were lower in the COPD group. The level of education was higher in subjects without COPD (Table 1).

### 3.2. Occupational Exposures and COPD

Most of the subjects (330, 62.5%) hadever been exposed to one or more of the occupational agents under study. The most frequent exposures were to biological dust, mineral dust and solvents. Subjects with COPD had higher percentages of exposure overall, specifically to biological dust, gases/fumes and aromatic and chlorinated solvents. COPD percentages were not compared for exposure to herbicides, insecticides or fungicides because of the low number of subjects exposed (Table 2). The jobs most frequently related to each occupational exposition are displayed in Table S1.

In a multivariate logistic regression model, adjusted for age, sex, education, biomass exposure, smoking status, and pack-years smoked, occupational exposure to VGDF was associated with COPD (OR 1.53; CI 0.93–2.53) and (2.25 CI 95% 1.19–4.22) for only low and ever high exposure to VGDF, respectively. For the other occupational agents, high exposure to biological dust and gases/fumes were associated with COPD. Low exposure to aromatic solvents, chlorinated solvents, other solvents, and metals were also associated with COPD.

### 3.3. Relationship between COPD and Radiological Variables

Emphysema detected by CT was higher in subjects with COPD compared to non-COPD subjects, both for the variable of the percentage of emphysema volume and in the 15th percentile of lung density. COPD subjects presented higher %WA values and lower values of the bronchial lumen area for both the primary and secondary bronchus compared to the non-COPD groups (Table 3 and Table S3).

### 3.4. Radiological Variables According to Occupational Exposure

In the adjusted linear regression models, low exposure to VGDF was significantly associated with the % of emphysema volume (coefficient 1.70, 95% CI 0.30–3.37); high exposure to gases/fumes and metals, exposure to chlorinated solvents and low exposure to aromatic solvents were associated with lower lung density defined by the 15th percentile (Table 4).

### 3.5. Relationship between Occupational Exposure and Radiological Variables with Respiratory Symptoms

Subjects exposed to VGDF and pesticides more frequently reported wheezing (*p* = 0.025 and *p* = 0.016, respectively). Subjects with expectoration had an elevated emphysema volume % and decreased lung density defined by the 15th percentile values (*p* = 0.0135 and *p* = 0.0241, respectively). Subjects with dyspnoea showed elevated values of the primary and secondary bronchial wall area (*p* = 0.0005 and *p* = 0.0063, respectively), as well as a decreased lumen area at both bronchial levels (*p* = 0.0016 and *p* = 0.0461, respectively). Wheezing individuals showed elevated primary and secondary bronchus wall area values (*p* = 0.003 and *p* = 0.0514, respectively) (Tables S1 and S2).

## 4. Discussion

In the present study, the relationship between exposure to specific occupational agents determined by JEM, COPD, and CT findings was analysed for the first time in the Spanish population. Exposure to VGDF and several specific agents has been found to be clearly associated with COPD. In addition, exposure to VGDF was associated with a higher emphysema volume and exposure to chlorinated, aromatic, or other solvents and metals was associated with a lower 15th percentile value of lung density.

The relationship between exposure to VGDF, biological and mineral dust, and gases/fumes and COPD has been studied by different authors in populations in Northern Europe, the United States and Asian countries [4,7,12,32,33,34]. The results of these studies do not coincide, probably due to differences in working conditions, the characteristics of the subjects, or the methods for assessing occupational exposure and diagnosing COPD. Regarding occupational exposure, the self-reported information in these studies is mostly generic and does not allow for an estimation of exposure to specific inhaled agents. In previous studies carried out with JEM, exposure to various substances has been associated with COPD, but there was no concordance among these studies either. Our results demonstrate that many occupational agents evaluated are associated with COPD in the Spanish population, such as VGDF, biological dust, gases/fumes and chlorinated and aromatic solvents. Although workers’ protection laws are already in force in Spain, our results further reinforce the need to use personal protective equipment in those occupational activities at risk of respiratory exposure.

The present study evaluated the relationship between occupational exposure, COPD and the pulmonary and bronchial alterations detected by CT. The increase in emphysema and the wall area, as well as the reduction in the bronchial lumen area, in subjects with COPD is consistent with the histological and radiological phenotypes described in this disease [35,36]. In most patients, both emphysema and bronchiolar alterations are caused by tobacco smoke, coexist to a greater or lesser degree, and contribute to chronic airflow obstruction.

A relevant contribution of the present study was the analysis of pulmonary alterations evaluated by CT in relation to specific occupational agents. Low exposure to VGDF was associated with a higher percentage of emphysema volume, while exposure to gases/fumes, chlorinated and aromatic solvents, and metals was associated with a lower 15th percentile of lung density. Both variables reflect low lung density and have been used to analyse emphysema in both cross-sectional and longitudinal studies. The percentage of emphysema volume with a density threshold at −950 HU is the classic indicator used in most studies, whereas the 15th percentile has been introduced more recently and is considered highly reproducible and sensitive to early changes in lung structure [37,38]. Taken together, our results suggest that occupational exposures are a risk factor for the development of emphysema as a core element of COPD. The identification of an association with low but not with high VGDF exposure has been previously observed in other studies. Several possible explanations have been proposed to understand this apparent paradox. The healthy worker effect would explain why highly exposed workers may leave their occupations earlier and have less cumulative exposure than workers involved in occupations with less exposure. In addition, low exposure may go unnoticed more easily and occur over longer periods of time, and thus, its effect could be greater and even be combined with other toxic agents. Moreover, some degree of exposure misclassification cannot be ruled out [39]. Regarding the associations with the 15th percentile of lung density, with the exception of gases/fumes, the rest of the exposures included a reduced number of subjects, and this fact may have influenced the finding of significant associations in only some exposures and not in others.

The finding that exposure to solvents, whether aromatic, chlorinated or otherwise, is associated not only with COPD but also with lower lung density, as detected by the 15th percentile of lung density, is, we believe, a relevant result. Solvents are part of various products, such as paints, petroleum, adhesives, pharmaceuticals and synthetic products, among many others, and are, therefore, widely used in the industrial sector. Initial studies in diverse populations have shown that solvent exposure is associated with nonspecific respiratory disease [40,41,42], and, with respect to bronchial obstruction, significant or nonsignificant associations have also been found [11,43,44]. These studies have important limitations, such as relying on self-reported exposure or on a definition of bronchial obstruction by pre-bronchodilator spirometry [42,43]. Subsequently, in a longitudinal study conducted in a Tasmanian population, Alif et al. demonstrated, for the first time, a robust association of chlorinated solvent exposure with fixed bronchial obstruction defined by post-bronchodilator spirometry in the female subgroup [44]. The data provided by the present study, therefore, reinforce the hypothesis that exposure to solvents is associated with an increased risk of pulmonary emphysema and poses a health hazard to our workers.

The association found between exposure to metals and lower lung density measured by the 15th percentile is also interesting. In classic studies, the inhalation of cadmium smoke had been associated with the development of emphysema [45]. Subsequently, studies in welders and steel workers reported an association with bronchial obstruction [46,47,48], while in population studies, there were discrepant results. In two studies conducted with prebronchodilator spirometry, no association was detected [43,49], while in a more recent study, a statistically significant association between an airway’s obstruction and a decrease in the diffusing capacity of the lungs for carbon monoxide was obtained [44]. These data suggest a relationship between metal exposure and the existence of emphysema. As far as we know, our results demonstrate, for the first time, the relationship between metal exposure and CT-detected emphysema.

This study has several limitations. First, its cross-sectional design precludes any causal inference between occupational exposures and COPD or CT findings. On the other hand, the CT scans were performed in each of the different centres with different devices, and while the image acquisition protocol was always the same, possible differences cannot be ruled out. However, this is a problem inherent to the multicentre nature of this study.

Likewise, this work has obvious strengths. In our opinion, the main strength was the determination of occupational exposure by means of the ALOHA(+) JEM, which allows the identification of a great number of occupational agents, and the diagnosis of COPD was confirmed by post-bronchodilator spirometry. Also noteworthy is the measurement of emphysema and primary and secondary bronchial areas according to the most recent methodology. Moreover, the adjustments introduced in the regression models were rigorous in order to minimise possible biases. Finally, the multicentre nature of this study makes it representative of the Spanish population as a whole.

## 5. Conclusions

The present study demonstrates that exposure to a good number of specific occupational agents is associated with the diagnosis of COPD determined by spirometry in the Spanish population. A relationship between various occupational exposures and radiological emphysema has also been demonstrated. These findings underline the urgent need to implement and reinforce measures for the prevention and control of exposures in the workplace to improve the respiratory health of our workers. Future studies should continue to explore effective interventions and mitigation strategies to protect the respiratory health of workers in various occupational settings.

## Figures and Tables

**Table 1:** Sociodemographic, lifestyle, and lung function characteristics of participants with and without COPD. EPISCAN II study, Spain 2017–2019.

Characteristics	COPD ^1^ (n = 226)	No COPD ^2^ (n = 300)	*p*-Value
Gender: n (%)			<0.001
Men	125 (55.3%)	111 (37.0%)	
Women	101 (44.7%)	189 (63.0%)	
Age (years)			
Mean (±SD)	66.6 (10.3)	60.2 (10.6)	<0.001
Range	42−89	40−88	
Body mass index: mean (±SD) ^3^	27.0 (4.4)	27.0 (4.8)	0.96
Smoking status: n (%)			<0.001
Current smoker	81 (35.8%)	60 (20.0%)	
Former smoker	97 (42.9%)	110 (36.7%)	
Non-smoker	48 (21.2%)	130 (43.3%)	
Pack-years smoked: mean (±SD)	30.8 (28.6)	13.9 (19.2)	<0.001
Biomass smoke exposure n (%)	43 (19.6%)	44 (15.2%)	0.191
Educational level: n (%)			0.023
No education	8 (3.6%)	2 (0.7%)	
Primary education	52 (23.2%)	57 (19.0%)	
Secondary education	49 (21.9%)	53 (17.7%)	
University, vocational training or similar	115 (51.3%)	187 (62.3%)	
Other or unknown	2 (0.9%)	1 (0.3%)	
FEV_1_ (% predicted): mean (±SD)	83.6 (17.6)	104.04 (14.8)	<0.001
FEV_1_/FVC (%): mean (±SD)	62.9 (7.9)	80.21 (5.3)	<0.001

^1^ FEV_1_ to FVC ratio < 70% tested in the main EPISCAN-II study and in a more detailed clinical survey. ^2^ FEV_1_ to FVC ratio ≥ 70% tested in the main EPISCAN-II study and in a more detailed clinical survey. ^3^ Missing information for 1 COPD case and 3 no COPD controls. Abbreviations: SD: standard deviation; COPD: chronic obstructive pulmonary disease; FEV_1_: forced expiratory volume in one second; and FVC: forced vital capacity.

**Table 2:** Associations between occupational exposures and COPD.

Agent	Level ^1^	COPD (n = 226)	No COPD (n = 300)	OR (95% CI) ^2^
Vapours, gases, dust or fumes	Low	89 (39.7%)	122 (40.8%)	1.53 (0.93–2.53)
	High	64 (28.6%)	52 (17.4%)	2.25 (1.19–4.22)
Biological dust	Low	77 (43.8%)	108 (43.4%)	1.68 (0.98–2.88)
	High	28 (15.9%)	16 (6.4%)	3.01 (1.22–7.45)
Mineral dust	Low	62 (37.6%)	60 (27.9%)	1.82 (0.99–3.34)
	High	32 (19.4%)	30 (14.0%)	1.96 (0.86–4.50)
Gases or fumes	Low	93 (44.3%)	123 (44.2%)	1.52 (0.91–2.54)
	High	46 (21.9%)	30 (10.8%)	2.49 (1.20–5.17)
Pesticides	Low	7 (8.3%)	7 (4.9%)	1.78 (0.45–7.15)
	High	6 (7.1%)	12 (8.3%)	1.35 (0.35–5.27)
Herbicides	Low	1 (1.3%)	1 (0.7%)	-- ^3^
	High	5 (6.5%)	12 (8.7%)	-- ^3^
Insecticides	Low	4 (5.0%)	2 (1.4%)	-- ^3^
	High	5 (6.3%)	11 (8.0%)	-- ^2^
Fungicides	Low	6 (7.2%)	7 (4.9%)	-- ^3^
	High	6 (7.2%)	11 (7.7%)	-- ^3^
Solvents	Low	61 (38.6%)	76 (34.5%)	1.68 (0.95–2.98)
	High	26 (16.5%)	19 (8.6%)	2.36 (0.92–6.03)
Aromatic solvents	Low	39 (33.9%)	35 (21.5%)	2.29 (1.03–5.08)
	High	5 (4.3%)	3 (1.8%)	5.21 (0.71–38.4)
Chlorinated solvents	Low	30 (25.9%)	25 (15.6%)	2.23 (1.00–4.97)
	High	15 (12.9%)	10 (6.3%)	3.39 (0.92–12.5)
Other solvents	Low	72 (46.5%)	77 (36.5%)	1.86 (1.06–3.28)
	High	12 (7.7%)	9 (4.3%)	2.25 (0.67–7.54)
Metals	Low	23 (19.5%)	27 (16.7%)	2.28 (0.90–5.80)
	High	24 (20.3%)	10 (6.2%)	2.87 (0.99–8.30)
Any of the above	Low/High	155 (68.6%)	175 (58.3%)	1.70 (1.09–2.64)
None of the above		71 (31.4%)	125 (41.7%)	1 (reference)

^1^ Low= only low exposure; high= ever high exposure; ^2^ Multivariate logistic regression models adjusted for age, sex, education, biomass exposure, smoking status, and pack-years smoked using no exposure to all agents as the uniform reference category (n = 196) ^3^ No comparisons were made due to the low number of exposed subjects. Abbreviations: OR: odds ratio; CI: confidence interval.

**Table 3:** Distribution of CT variables according to COPD and smoking status.

Variable	No COPD Never Smokers (n = 130)	No COPD Ever Smokers (n = 170)	COPD Never Smokers (n = 48)	COPD Ever Smokers (n = 178)	*p*-Value
% Total Emphysema Volume (Fixed Threshold)	3.53 (4.67)	4.52 (6.50)	7.77 (6.00)	10.02 (11.86)	<0.001
15th percentile lung density	−912.04 (24.27)	−911.64 (30.37)	−929.17 (20.51)	−930.82 (25.12)	<0.001
%Airway Wall Area Primary Bronchi	48.82 (6.01)	48.36 (5.52)	53.56 (5.25)	52.09 (5.22)	<0.001
%Airway Wall Area Secondary Bronchi	62.25 (8.00)	62.32 (8.16) ^1^	68.17 (6.17)	66.85 (6.62)	<0.001
Lumen Area–Primary Bronchi	43.08(20.83)	43.77 (19.80)	29.63 (11.65)	33.77 (14.00)	<0.001
Lumen Area–Secondary Bronchi	14.43 (9.34)	14.29 (9.62) ^1^	8.27 (3.64)	9.84 (5.85)	<0.001

Mean (standard deviation) of each variable is presented in each subgroup ^1^ Missing information for 1 individual. Abbreviations: COPD: chronic obstructive pulmonary disease.

**Table 4:** Associations between occupational exposures and the 15th percentile of lung density.

Agent	Level	Coefficient (95% CI) ^1^	*p*-Value
Vapours, gases, dust or fumes	Low	−2.58 (−7.77; +2.61)	
	High	+6.05 (−0.47; +12.57)	
Biological dust	Low	+0.38 (−4.82; +5.59)	
	High	−0.26 (−8.94; +8.42)	
Mineral dust	Low	+0.86 (−5.58; +7.30)	
	High	+6.90 (−1.58; +15.38)	
Gases or fumes	Low	−1.42 (−6.61; +3.76)	
	High	+8.65 (+1.21; +16.09)	<0.05
Pesticides	Low	−2.31 (−15.72; +11.11)	
	High	−2.30 (−14.91; +10.30)	
Herbicides	Low	−13.87 (−47.68; +19.93)	
	High	−3.20 (−16.35; +9.96)	
Insecticides	Low	−4.71 (−25.10; +15.67)	
	High	−1.43 (−14.90; +12.04)	
Fungicides	Low	−1.93 (−15.86; +12.00)	
	High	−1.03 (−13.95; +11.89)	
Solvents	Low	+2.62 (−3.04; +8.27)	
	High	+7.15 (+1.86; +16.16)	
Aromatic solvents	Low	+8.43 (+1.16; +15.70)	<0.05
	High	+11.64 (−6.25; +29.54)	
Chlorinated solvents	Low	+8.76 (+1.08; +16.44)	<0.05
	High	+11.91 (+0.46; +23.36)	<0.05
Other solvents	Low	+3.56 (−2.03; +9.15)	
	High	+2.68 (−9.42; +14.78)	
Metals	Low	+0.47 (−7.84; +8.78)	
	High	+14.45 (+4.42; +24.49)	<0.05
None of the above		1 (reference)	

^1^ Multivariate linear regression models adjusted for age, sex, education, biomass exposure, smoking status, pack-years smoked and COPD using no exposure to all agents as the uniform reference category (n = 196). Abbreviations: COPD: chronic obstructive pulmonary disease; CI: confidence interval

## Data Availability

Please refer to the GSK weblink to access GSK’s data-sharing policies and, as applicable, seek anonymised subject-level data via the link https://www.gsk-studyregister.com/en/.

## Supplementary Materials

The following supporting information can be downloaded at https://www.mdpi.com/article/10.3390/toxics12100689/s1. Table S1: Most common reported occupations for the main exposure categories, Table S2: Respiratory symptoms according to exposure to vapours, gases, dusts, fumes, and pesticides, Table S3: CT variables according to respiratory symptoms.
