# Supplementary material for: Occupational Exposures, Chronic Obstructive Pulmonary Disease and Tomographic Findings in the Spanish Population
Source: Toxics. 2024 Sep 24;12(10):689. doi: 10.3390/toxics12100689 (PMC11510821; doi:10.3390/toxics12100689)
Supplement: Supplementary file 1 [file toxics-12-00689-s001.zip › toxics-3164651-supplementary.pdf]

Table S1: Most common reported occupations for the main exposure categories

Agent	Level	Number[1]	Most recorded occupations (ISCO-88 code)[2]
Vapours, gases, dust or fumes	Low	428	Armed forces (110); nurses (2230); other health professionals (222); cooks (5122); personal care workers (513); hairdressers (5141); bricklayers and stonemasons (7122); tailors (7433); car, taxi and van drivers (8322); cleaners (9132).
	High	161	Welders (7212); painters (7141); machine-tool setters and setter-operators (7223); heavy-truck and lorry drivers (8324); farm-hands and labourers (9211).
Pesticides	Low/High	32	Field crop and vegetable growers (6111); dairy and livestock producers (6121); farm-hands and labourers (9211).
Solvents	Low	174	Medical doctors (2221); nurses (2230); personal care workers (513).
	High	48	Hairdressers (5141); painters (7141); machine-tool setters and setter-operators (7223); motor vehicle mechanics and fitters (7231).
Metals	Low/High	112	Physical and engineering science technicians (311); painters (7141); welders (7212); machine-tool setters and setter-operators (7223); assembling labourers (9321).
No exposure (none of the above)	-	503	Housewives (-); teachers (233); technical and commercial sales representatives (3415); administrative workers (3431); office clerks (41); shop salespersons and demonstrators (5220).

[1] Total number of reported jobs that were classified by the JEM in this exposure category. In total, 1096 jobs were reported by the 526 study participants. Different exposures may overlap.
[2] An occupational description for all ISCO-88 codes can be found at https://ilostat.ilo.org/methods/concepts-and-definitions/classification-occupation/

Table S2. Respiratory symptoms according to exposure to vapours, gases, dusts and fumes and pesticides.

Variable		None	Low	High	p-value
		**Vapours, gases, dust or fumes**			
Chronic cough	Total	191 (100.0%)	208 (100.0%)	114 (100.0%)	0.4659
	No	150 (78.5%)	169 (81.3%)	86 (75.4%)	
	Yes	41 (21.5%)	39 (18.8%)	28 (24.6%)	
	N missings	5	3	2	
Chronic bronchitis	Total	177 (100.0%)	199 (100.0%)	110 (100.0%)	0.0541
	No	163 (92.1%)	189 (95.0%)	96 (87.3%)	
	Yes	14 (7.9%)	10 (5.0%)	14 (12.7%)	
	N missings	19	12	6	
Expectoration	Total	195 (100.0%)	210 (100.0%)	115 (100.0%)	0.5022
	No	161 (82.6%)	175 (83.3%)	90 (78.3%)	
	Yes	34 (17.4%)	35 (16.7%)	25 (21.7%)	
	N missings	1	1	1	
Dyspnea	Total	191 (100.0%)	208 (100.0%)	114 (100.0%)	0.2140
	No	164 (85.9%)	173 (83.2%)	89 (78.1%)	
	Yes	27 (14.1%)	35 (16.8%)	25 (21.9%)	
	N missings	5	3	2	
Wheezing	Total	196 (100.0%)	210 (100.0%)	116 (100.0%)	**0.0253**
	No	127 (64.8%)	132 (62.9%)	58 (50.0%)	
	Yes	69 (35.2%)	78 (37.1%)	58 (50.0%)	
	N missings	0	1	0	

Table S2. Cont.

Variable		None	Low	High	p-value
			**Vapours, gases, dust or fumes**		
Any symptom (ECCS)	Total	190 (100.0%)	209 (100.0%)	112 (100.0%)	0.2279
	No	83 (43.7%)	95 (45.5%)	40 (35.7%)	
	Yes	107 (56.3%)	114 (54.5%)	72 (64.3%)	
	N missings	6	2	4	
**Variable**		**None**	**Low**	**High**	**p-value**
			**Pesticides**		
Chronic cough	Total	191 (100.0%)	14 (100.0%)	18 (100.0%)	0.5818
	No	150 (78.5%)	11 (78.6%)	16 (88.9%)	
	Yes	41 (21.5%)	3 (21.4%)	2 (11.1%)	
	N missings	5	0	0	
Chronic bronchitis	Total	177 (100.0%)	14 (100.0%)	17 (100.0%)	0.6596
	No	163 (92.1%)	12 (85.7%)	16 (94.1%)	
	Yes	14 (7.9%)	2 (14.3%)	1 (5.9%)	
	N missings	19	0	1	
Expectoration	Total	195 (100.0%)	14 (100.0%)	18 (100.0%)	0.8293
	No	161 (82.6%)	12 (85.7%)	14 (77.8%)	
	Yes	34 (17.4%)	2 (14.3%)	4 (22.2%)	
	N missings	1	0	0	
Dyspnea	Total	191 (100.0%)	13 (100.0%)	17 (100.0%)	0.4435
	No	164 (85.9%)	12 (92.3%)	13 (76.5%)	
	Yes	27 (14.1%)	1 (7.7%)	4 (23.5%)	
	N missings	5	1	1	
Wheezing	Total	196 (100.0%)	14 (100.0%)	18 (100.0%)	**0.0157**
	No	127 (64.8%)	4 (28.6%)	9 (50.0%)	
	Yes	69 (35.2%)	10 (71.4%)	9 (50.0%)	

Abbreviations: European Coal and Steel Community (ECCS) questionnaire on respiratory symptoms

Table S3. CT variables according to respiratory symptoms.

Variable		No	Yes	p-value
			**Expectoration**	
% Total Emphysema Volume (Fixed Threshold)	Valid n	429	94	**0.0135**
	Mean(± SD)	6.01 (8.11)	8.49 (11.32)	
Percentil 15	Valid n	429	94	**0.0465**
	Mean(± SD)	-918.79 (26.90)	-925.12 (31.78)	
%Airway Wall area Primary Bronchi	Valid n	429	94	0.7151
	Mean(± SD)	50.13 (5.78)	50.37 (6.04)	
%Airway Wall area Secondary Bronchi	Valid n	429	93	0.7310
	Mean(± SD)	64.33 (8.04)	64.64 (6.86)	
Lumen area - Primary Bronchi	Valid n	429	94	0.4502
	Mean(± SD)	38.72 (17.18)	40.31 (23.44)	
Lumen area - Secondary Bronchi	Valid n	429	93	0.7864
	Mean(± SD)	12.23 (8.34)	12.49 (8.61)	

Table S3. Cont.

Variable		No	Yes	p-value
		**Dyspnoea**		
% Total Emphysema Volume (Fixed Threshold)	Valid n	429	87	0.2811
	Mean(± SD)	6.31 (8.39)	7.44 (10.86)	
Percentil 15	Valid n	429	87	0.7027
	Mean(± SD)	-920.31 (27.00)	-919.06 (32.48)	
%Airway Wall area Primary Bronchi	Valid n	429	87	**0.0005**
	Mean(± SD)	49.83 (5.75)	52.21 (5.91)	
%Airway Wall area Seconary Bronchi	Valid n	429	86	**0.0063**
	Mean(± SD)	64.01 (7.84)	66.54 (7.66)	
Lumen area - Primary Bronchi	Valid n	429	87	**0.0016**
	Mean(± SD)	40.05 (18.92)	33.21 (15.22)	
Lumen area - Secondary Bronchi	Valid n	429	86	**0.0461**
	Mean(± SD)	12.55 (8.60)	10.58 (6.88)	
		**Wheezing**		
% Total Emphysema Volume (Fixed Threshold)	Valid n	320	205	0.3512
	Mean(± SD)	6.13 (8.34)	6.86 (9.48)	
Percentil 15	Valid n	320	205	0.5662
	Mean(± SD)	-919.22 (27.65)	-920.65 (28.30)	
%Airway Wall area Primary Bronchi	Valid n	320	205	**0.0172**
	Mean(± SD)	49.73 (5.77)	50.98 (5.90)	
%Airway Wall area Secondary Bronchi	Valid n	320	204	**0.0371**
	Mean(± SD)	63.80 (7.89)	65.26 (7.68)	
Lumen area - Primary Bronchi	Valid n	320	205	0.0514
	Mean(± SD)	40.15 (18.82)	36.93 (17.71)	
	Valid n	320	204	0.2243
Lumen area - Secondary Bronchi	Mean(± SD)	12.63 (8.41)	11.72 (8.28)	

Mean and standard deviation (SD) of each variable is presented in each subgroup
